# Microglial TREM2 Mitigates Inflammatory Responses and Neuronal Apoptosis in Angiotensin II-Induced Hypertension in Middle-Aged Mice

**DOI:** 10.3389/fnagi.2021.716917

**Published:** 2021-08-20

**Authors:** Xiaotian Xu, Lin Du, Jianxiong Jiang, Ming Yang, Zhaoxia Wang, Yingge Wang, Tieyu Tang, Xuetao Fu, Jiukuan Hao

**Affiliations:** ^1^Department of Neurology, The Affiliated Hospital, Yangzhou University, Yangzhou, China; ^2^Department of Pharmacological & Pharmaceutical Sciences, College of Pharmacy, University of Houston, Houston, TX, United States; ^3^Department of Cardiology, The Affiliated Hospital, Yangzhou University, Yangzhou, China; ^4^Department of Pharmaceutical Sciences, College of Pharmacy, University of Tennessee Health Science Center, Memphis, TN, United States

**Keywords:** LOAD, hypertension, aging, TREM2, microglia, astrocyte, neuroinflammation

## Abstract

Growing evidence suggests that hypertension and aging are prominent risk factors for the development of late-onset Alzheimer’s disease (LOAD) by inducement of neuroinflammation. Recent study showed that neuroinflammation *via* activated microglia induces reactive astrocytes, termed A1 astrocytes, that highly upregulate numerous classical complement cascade genes that are destructive to neurons in neurodegeneration diseases. Moreover, triggering receptor expressed on myeloid cells 2 (TREM2) is considered as one of the strongest single-allele genetic risk factors and plays important roles in neuroinflammation for LOAD. However, the mechanisms of microglia in the regulation of A1 astrocytic activation are still not clear. We introduced angiotensin II-induced hypertension in middle-aged mice and found that hypertension-upregulated TREM2 expression and A1 astrocytic activation were involved in neuroinflammation in the animal models used in this study. The *in vitro* results revealed that overexpression of microglial TREM2 not only mitigated microglial inflammatory response but also had salutary effects on reverse A1 astrocytic activation and neuronal toxicity.

## Introduction

Alzheimer’s disease is the most common cause of all types of dementia (Lane et al., 2018; Scheltens et al., 2021), and sporadic Alzheimer’s disease (AD) usually occurs after the of 65, and is also named late-onset AD (LOAD) (Rabinovici, 2019). Growing evidence suggests that hypertension plays an important role in LOAD (Kruyer et al., 2015; Lüders and Schrader, 2015) by causing Aβ plaque deposits and cerebral amyloid angiopathy (CAA) (Tsukuda et al., 2009; Carnevale et al., 2012; Elias et al., 2012). Furthermore, clinical research investigators have confirmed that midlife hypertension is strongly correlated with late-life dementia in humans (Iadecola, 2014), and that effective treatment for hypertension in midlife can attenuate the risk of developing cognitive impairment in older age (Elias et al., 2012; Tzourio et al., 2014; Baranowski et al., 2018).

Although the mechanisms of hypertension causing dementia are still not fully understood, it has been found that neuroinflammation plays a pivotal role in the incidence and progression of AD (Iadecola, 2014; McMaster et al., 2015; Bolos et al., 2017). As inflammatory response cells in the brain, microglia play important roles in immune responses in the central nervous system and is involved in the pathogenesis of neurodegenerative and neuroinflammatory diseases. In response to inflammation, microglia can be activated and polarized into pro-inflammatory M1 phenotype or anti-inflammatory M2 phenotype. The M1 activation of microglia occurs in response to Aβ and other inflammatory stimuli, which have detrimental impacts on neurons, through the release of pro-inflammatory factors and various toxic substances (Sarlus and Heneka, 2017; Hansen et al., 2018).

A recent study has shown that M1 microglia activated astrocytes into A1 astrocytes, which highly upregulate numerous classical complement cascade genes that are destructive to neurons and oligodendrocytes in neurodegenerative diseases (Liddelow et al., 2017). Blocking M1 microglial-induced A1 astrocytic activation is considered to be an effective therapeutic strategy for AD (Baldwin and Eroglu, 2017; Clarke et al., 2018). Notably, TREM2 plays important roles on microglial functions such as phagocytosis, biosynthetic metabolism, and inflammatory response (Condello et al., 2018), and TREM2 deficiency exacerbates activated M1 microglial inflammatory cytokines release and neuronal apoptosis, but TREM2 overexpression markedly attenuated inflammation and neuronal death in AD model studies (Jiang et al., 2014; Jay et al., 2015, 2017). Furthermore, genetic studies unveil that rare coding variants in TREM2 in microglia increase the risk of developing LOAD by 3–4 folds (Jin et al., 2014; Song et al., 2017; Zhong et al., 2019). Although TERM2 is considered as one of the strongest single-allele genetic risk factors for LOAD (Jiang et al., 2016), its potential roles in the onset and progression of the disease still remain to be explored.

In this study, we introduced hypertension in middle-aged mice to investigate the mechanisms in the progression of LOAD. We found that hypertension upregulated TREM2 expression and A1 astrocytes in middle-aged mice for the first time. Based on the anti-neuroinflammatory role of TREM2 that has been proven in AD, in this study, we hypothesized whether TREM2 upregulation in microglia could be a potential strategy to impede M1 microglial-induced A1 astrocytes and neuronal toxicity. We then performed *in vitro* experiments and revealed that the overexpression of microglial TREM2 not only mitigated microglial inflammatory response, but also had salutary effects on reverse A1 astrocytic activation and neuronal toxicity.

## Materials and Methods

### Materials

#### Chemicals

Lipopolysaccharides (LPS; L2880) and the anti-glial fibrillary acidic protein (anti-GFAP; G9269) antibody were purchased from Sigma (St. Louis, MO, United States). The complement C3 polyclonal antibody (sc-58926) was purchased from Santa Cruz Biotechnology (Dallas, TX, United States). Dulbecco’s Modified Eagle’s Medium (DMEM), fetal bovine serum (FBS), phosphate-buffered saline (PBS), and penicillin-streptomycin, 4′,6-diamidino-2-phenylindole (DAPI) were purchased from Life Technologies (Carlsbad, CA, United States). Anti-tubulin antibodies (2148S) were purchased from Cell Signaling Technology (Beverly, MA, United States). Goat anti-rabbit IgG H&L (Alexa Fluor 488), rabbit anti-rat IgG H&L (Alexa Fluor 555), TNF-α enzyme-linked immunosorbent assay (ELISA) kits, and IL-1α ELISA kits were purchased from Abcam (San Francisco, CA, United States). C1q ELISA Kit (mouse; HK211) was purchased from Hycult Biotech (Plymouth Meeting, PA, United States). The anti-aquaporin 4 (AQP4) (249-323) antibody (AQP-004) was purchased from Alomone Labs (Jerusalem, Israel). The TREM2 polyclonal antibody (PA5-87933) and lactate dehydrogenase (LDH) release were purchased from Thermo Fisher Scientific (Waltham, MA, United States). Cell Counting Kit-8 was purchased from Dojindo Molecular Technologies (Rockville, MD, United States).

#### Microglia BV2 Cell Line Culture, Treatment, and Transfection, and Conditioned Medium Preparation

In this study, BV2 cells were cultured in DMEM with 10% FBS and penicillin-streptomycin at 37°C with 5% CO2. Then, the BV2 cells were pretreated with serum-free DMEM containing LPS (0.1 ug/ml). After incubation for 24 h, the conditioned medium was collected and denoted as MCM. Meanwhile, the BV2 cell complete culture medium was denoted as MCM-control. The BV2 cells (4 × 10^5^ cells per well) were seeded onto six-well plates overnight to reach 70% confluence for transfection. TREM2 (Myc-DDK-tagged) overexpression plasmid (MR202717) or pCMV6-Entry Mammalian Expression Vector (PS100001), which was purchased from OriGene Technologies, Inc. (Rockville, MD, United States), was transfected into cells for 4.5 h using Lipofectamine 3000 (Thermo Fisher Scientific, Waltham, MA, United States) and then replaced with a complete medium to culture for another 48 h at 37°C, which was then collected for use in subsequent experiments. The C-Myc mouse monoclonal antibody (TA500002, OriGene Technologies, Inc., Rockville, MD, United States) was applied to confirm transduction efficiency. For combination treatments, LPS (0.1 ug/ml) was added to the BV2 cells after TREM2 (vector or overexpression) transfection for 24 h, and culturing was continued for another 24 h at 37°C. The above conditioned mediums containing LPS (0.1 ug/ml) and TREM2 (vector or overexpression) in BV2 cells were collected and were denoted as MCM + TREM2 (vector or overexpression).

#### Primary Astrocyte Cultures, Treatment, and Staining

Primary cortical astrocyte cultures were prepared using 1- to 3-day-old neonatal c57mouse brains as previously described (Schildge et al., 2013), which were obtained from Charles River Laboratories (Wilmington, MA, United States). Briefly, the mice were decapitated, the cerebral cortices were removed, and the meninges were carefully stripped off. Tissues were maintained in DMEM and nutrient mixture F12 (DMEM/F12) and dissociated into single cells in the DMEM/F12 medium supplemented with 10% FBS. The cultures were incubated at 37°C in a humidified 5% CO2, 95% air atmosphere. The cell culture medium was changed 24 h after plating and, subsequently, every 3 days, until confluence was reached, which usually occurs after 7–10 days. The flasks were shaken at 180 rpm for 30 min in an orbital shaker to remove microglia, and the supernatant containing microglia was discarded. Afterward, a 20-ml fresh astrocyte culture medium was added to the flakes, and the experiment was continued by shaking the flask at 240 rpm for 6 h to remove oligodendrocyte precursor cells. After that, cells were subjected to a passage to generate purified astrocyte cultures (secondary cultures), which constituted more than 95% of GFAP-positive cells. For the treatments, first, the astrocytes were treated with MCM to induce A1 astrocytic activation. After incubation for 24 h, the conditioned medium was collected and denoted as ACM. Second, the astrocytes were treated with MCM + TREM2 (vector or overexpression) for 24 h, and the above conditioned mediums containing MCM + TREM2 (vector or overexpression) in astrocytes were collected and denoted as ACM + TREM2 (vector or overexpression). For immunocytochemistry, the astrocytes were seeded at 0.8 × 106 on 1.5-mm^2^ coverslips for 24 h. After treatment, the cells were fixed with 4% ice-cold paraformaldehyde for 20 min at 4°C and air dried. Then, blocking and permeabilization were performed with 1% BSA in PBS with.1% Triton for 30 min. After that, the astrocyte (A1) was stained with the C3 antibody (1:1,000) and GFAP antibodies (1:2,000) or the AQP4 antibody (1:500) for 24 h in a humidified chamber at 4°C. After three washes with.1% Triton in PBS, goat anti-rat IgG H&L (Alexa Fluor 555, 1:200) and goat anti-rabbit IgG H&L (Alexa Fluor 488, 1:200) was used for 1 hr. After DAPI staining for 10 min, the coverslips were transferred onto glass slides. Images were obtained using a digital microscope camera system (Nikon DS-Ri2, Nikon, Tokyo, Japan).

#### Cell Viability Assay

Murine neuronal-like (Neuro2a) cell line is widely used the neuronal cell line for investigation of neuronal survival, neuronal differentiation, and neuroprotection *in vitro* (Eom et al., 2015; Nicolas et al., 2015; Steiner et al., 2016). In this study, the viability of Neuro2A cells following incubation with MCM, ACM, TREM2 overexpression + MCM, and TREM2 overexpression + ACM was evaluated by lactate dehydrogenase (LDH) release and Cell Counting Kit-8 (CCK-8) assays. The amount of LDH released was expressed as a percentage of the value obtained in comparative wells where cells were 100% lysed by 1% Triton X-100. For the CCK-8 assays, data are presented as a percentage of the value obtained from cells incubated in a fresh medium only.

#### Animals

This study was approved by the Animal Research Committee of Sun Yat-sen University (Guangzhou, China; Committee Reference Number: SYSU-IACUC-2018-000093). All efforts were made to minimize the number and suffering of animals used in this study. Fourteen male C57BL/6J mice (30–35 g) obtained from Beijing Vital River Laboratory Animal Technology Co., Ltd. (Stock Number: SCXK2016-0006) were used. All the animals were used at 10 months of age (considered middle age in mouse), housed under a 12:12 h light-dark cycle (light from 07:00 to 19:00) with controlled temperature and humidity, and given food and water *ad libitum*.

#### Hypertension in Mouse Model Induced by Chronic Angiotensin Infusion

The hypertension mouse model was established as described previously (Gentile et al., 2009). Briefly, angiotensin II (AGT II,0.5 ng/kg/day in.9% NaCl, Sigma-Aldrich, St. Louis, MO, United States) was infused into a group of seven mice for 30 days with an osmotic mini pump (Durect, Cupertino, CA, United States) implanted subcutaneously. An additional group of seven mice was infused with saline vehicle alone as a control. Blood pressure was measured with the tail-cuff method on days 0, 7, 14, 21, and 30 during the 30-day infusion period.

#### Histology

The mice were perfused with 50 ml of ice-cold.9% saline followed by 50 ml of 4% (w/v) paraformaldehyde in phosphate-buffered saline (PBS; pH 7.4). Their brains were removed and incubated overnight in 4% paraformaldehyde and then dehydrated in 20–30% sucrose in PBS. Coronal brain slices (14-mm thick) from the right parietal cortex were sectioned with a frozen microtome (Leica Biosystems, Wetzlar, Germany) to produce consecutive frozen sections. For immunofluorescence staining, the sections were boiled in a citric acid buffer (pH 6) for 5 min in a microwave oven. After the sections were cooled, they were treated with.3% Triton X-100 and 10% goat serum for 1 h at room temperature. The sections were then incubated overnight at 4°C with a primary antibody (1:100 anti-ionized calcium binding adapter molecule 1, IBA1) antibody, catalog number 019-19741, Wako; 1:100 anti-glial fibrillary acidic protein (GFAP) antibody, catalog number AMAb91033, Sigma-Aldrich (St. Louis, MO, United States); 1:50 anti-amyloid antibody, β1-40, catalog number AB5074P, Millipore (Burlington, MA, United States); 1:50 anti-amyloid antibody, β1-42, catalog number AB5078P, Millipore (Burlington, MA, United States); 1:100 anti-c3 antibody, catalog number sc-58926, Santa Cruz Biotechnology (Dallas, TX, United States); or 1:200 anti-CD68 antibody, catalog number ab125212, Abcam (San Francisco, CA, United States), and then incubated for 1 h at room temperature with a secondary antibody (1:500 goat anti-rabbit IgG H&L, Alexa Fluor^®^ 488), catalog number ab150077, Abcam (San Francisco, CA, United States); 1:500 goat anti-mouse IgG H&L (Alexa Fluor^®^ 555), catalog number ab150118, Abcam (San Francisco, CA, United States); or 1:500 rabbit anti-goat IgG H&L (Alexa Fluor^®^ 555), catalog number ab150146, Abcam (San Francisco, CA, United States) in PBS containing 10% blocking solution. The sections were mounted onto slides, stained with DAPI solution with antifade (Sigma-Aldrich, St. Louis, MO, United States), and covered with a coverslip. To evaluate the Aβ expression levels, the percentage of the Aβ40 or the Aβ42 plaque area was measured within the cortex and hippocampus. The percentage of GFAP-positive astrocytes with C3 localization among total GFAP-positive astrocytes was determined from the images. A similar method was applied to analyze other positive markers of target cells.

#### TUNEL Assay

The terminal deoxynucleotidyl transferase dUTP nick end labeling staining method is used to detect fragments of DNA in apoptotic cells in tissue samples (Kyrylkova et al., 2012). In this study, TUNEL staining was performed to detect DNA fragments during neuronal apoptosis. A TUNEL assay was performed according to the directions of the manufacturer using *In Situ* Cell Death Detection Kit (CAT: 11684817910, Roche, Indianapolis, IN, United States). In brief, the sections were washed three times for 10 min each. Then, the sections were incubated in.3% Triton-X and 0.1% sodium citrate and rinsed three times with PBS for 10 min each. The sections were incubated in.3% H2O2 in PBS for 30 min and then rinsed with PBS. The sections were then incubated with a 50-ml TUNEL reaction mixture in a humidified atmosphere for 60 min at 37°C in the dark. Then, the sections were rinsed three times with PBS. The sections were then observed under a digital microscope camera system (Nikon DS-Ri2, Nikon, Tokyo, Japan). A negative control was carried out by incubating the sections in 50 ml of a label solution without terminal transferase instead of the TUNEL reaction mixture. 4,6-Diamino-2-phenyl indole (DAPI, Sigma-Aldrich, St. Louis, MO, United States) was used for nuclear staining, and a primary antibody (anti-NeuN antibody [EPR12763], ab177487) was used for neuronal staining. To quantify the degree of apoptotic neuron death in the cortex and hippocampus, the percentage of cells labeled with both TUNEL-positive fluorescein and NeuN localized in the DAPI-stained nucleus out of the total number of NeuN-positive cells was determined from the images.

#### ELISA

The levels of C1q, TNF-α, and IL-1α were determined in the cell-culture medium obtained from the experiments. The culture medium was analyzed with a commercially ELISA kit according to the protocol of the manufacturer. Analysis of optical density or fluorescence was performed in a plate reader.

#### Western Blot Analysis

Cells were scraped and lysed in a RIPA lysis buffer on ice after treatment of 1 h at 4°C. Protein was extracted and quantified using a BCA assay kit (Thermo Scientific, Waltham, MA, United States) according to the instructions of the manufacturer. The cell lysates were solubilized in a sodium dodecyl sulfate (SDS) sample buffer (40 μg/lane) and separated by 10% SDS-polyacrylamide gel electrophoresis (110 V for 75 min). After electrophoresis, the protein was transferred to polyvinylidene difluoride (PVDF) membranes. Then, the membranes were blocked with 3% bovine serum albumin (BSA) and incubated with primary antibodies overnight at 4°C, followed by a horseradish peroxidase-conjugated secondary antibody for 1 h at room temperature, and detected with the enhanced chemiluminescence plus detection system (Millipore, Billerica, MA, United States). The density of each band was quantified using the Quantity One image analysis software (Bio-Rad, Life Science, Hercules, CA, United States).

#### Data and Statistical Analyses

The ImageJ software (National Institutes of Health, Bethesda, MD, United States) was used to analyze the immunofluorescence results. To evaluate the immunofluorescence results, the number of target cells was counted using the ImageJ software. The significance of differences in data sets was analyzed by two-tailed Student’s *t*-test or one-way analysis of variance tests. The data were expressed as the mean ± SD and analyzed using the GraphPad Prism 6 software (GraphPad, La Jolla, CA, United States). A *p* value < 0.05 was considered statistically significant.

## Results

### Hypertension Exacerbates β-Amyloid Deposition and Causes Neuronal Apoptosis in Middle-Aged Mice

**FIGURE 1:**
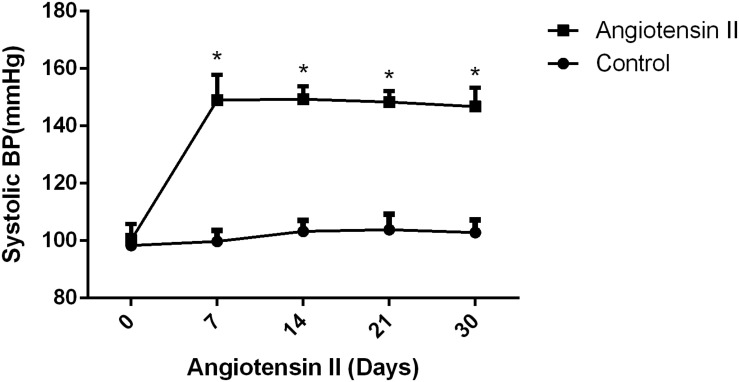
Effect of angiotensin II on systolic blood pressure (SBP) in middle-aged mice. The systolic blood pressure of the middle-aged mice was measured 7, 14, 21, and 30 days after the infusion of saline or angiotensin II (*n* = 7 per group). Data are expressed as the means ± SD. * *p* < 0.05.

**FIGURE 2:**
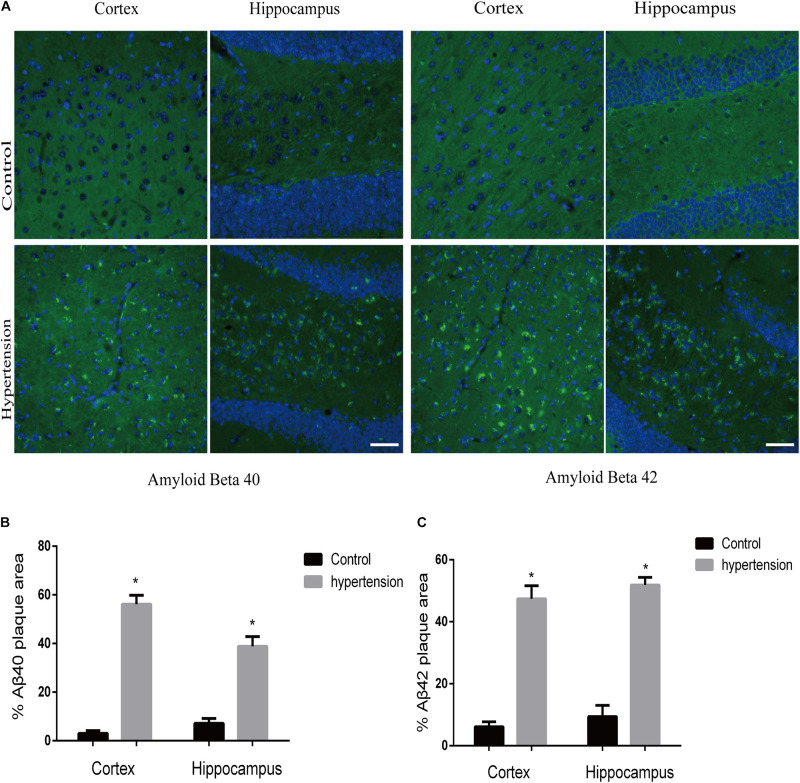
Aβ40 and Aβ42 deposition in the brain. After 30 days of angiotensin II treatment in middle-aged mice, the brain slices were subjected to immunofluorescence staining with the anti-Aβ40 (green) or the anti-Aβ42 (green) antibody and DAPI (blue) to observe β-amyloid deposition in the cortex and hippocampus. **(A)** Representative images of Aβ40 or Aβ42 deposition in the control and hypertension groups (immunofluorescence, ×40, scale bar = 50 μm). **(B,C)** Histograms comparing the percentage of Aβ40 or Aβ42 plaque area in the cortex and hippocampus of the control and hypertension groups (*n* = 7 per group). Data are expressed as the means ± SD. * *p* < 0.05.

**FIGURE 3:**
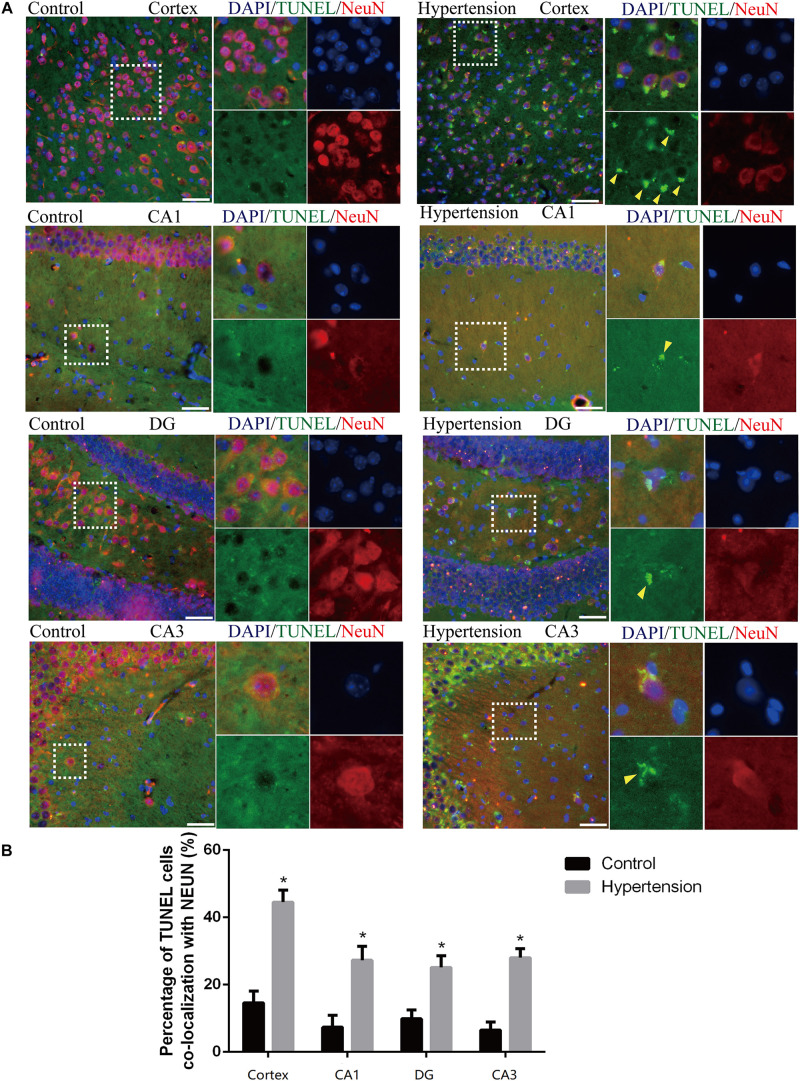
Angiotensin II-induced hypertension promotes neuronal apoptosis in middle-aged mice. **(A)** Representative images of TUNEL-positive, fluorescein-labeled (green) neurons co-stained with the anti-NeuN antibody (red) in the cortex and hippocampus (CA1, DG, and CA3) of mice in the control and hypertension groups (immunofluorescence, ×20, scale bar = 50 μm). **(B)** Histograms comparing the level of positive TUNEL staining in neurons in the cortex and hippocampus (CA1, DG, and CA3) of mice in the control and hypertension groups. Data are expressed as the means ± SD. * *p* < 0.05. (Negative control image of TUNEL staining assay provided in Supplementary Figure 3).

We first established hypertension, induced by angiotensin II, in the middle-aged mouse models. Systolic blood pressure (SBP) was measured on 0(98 ± 3.16 vs. 100 ± 5.51, *p* = 0.47), 7(99.5 ± 3.81 vs. 148.14 ± 8.45, *p* < 0.01), 14 (103.57 ± 3.69 vs. 149.42 ± 4.49, *p* < 0.01), 21(104.57 ± 5.01 vs. 147.42 ± 2.77, *p* < 0.01), and 30(103.28 ± 4.19 vs. 147.71 ± 5.96, *p* < 0.01) days during angiotensin II or saline (vector) osmotic mini pump administration. As shown in Figure 1, chronic infusion of angiotensin II with an osmotic mini pump evokes a significant increase in SBP, which reaches its peak at the seventh day of administration compared with the mice infused with saline. The hypertension effect was stable and continuous throughout the rest of the 30-day infusion period. Once hypertension was established, we assessed the effect of angiotensin II on Aβ deposition in the brain of the mice. As shown in Figure 2, the depositions of Aβ40 (3 ± 0.01% vs. 56% ± 0.04%, *p* < 0.01, cortex; 7 ± 0.02% vs. 39% ± 0.04, *p* < 0.01, hippocampus) and Aβ42 (6 ± 0.02% vs. 47 ± 0.04%, *p* < 0.01, cortex; 9 ± 0.03% vs. 52 ± 0.03%, *p* < 0.01, hippocampus) are significantly higher in the cortex and hippocampus of the mice in the hypertension group compared with the control group. Furthermore, neuronal apoptosis in the cortex and hippocampus was evaluated by TUNEL assay (Figure 3). The results revealed that hypertension markedly increased neuronal apoptosis in the cortex (13.71 ± 2.49% vs. 44.42 ± 3.86%, *p* < 0.01) and hippocampus (6.42 ± 2.50% vs. 27.57 ± 4.35%, *p* < 0.01, DG; 10.57 ± 1.9% vs. 24.28 ± 2.81%, *p* < 0.01, CA1; 6.57 ± 2.63% vs. 27.57 ± 2.63%, *p* < 0.01, CA3) of the mice in the angiotensin II infusion group compared with the mice in vehicle control.

### Neuroinflammatory Network Under Hypertension in Middle-Aged Mice

#### M1 Microglia Activation

**FIGURE 4:**
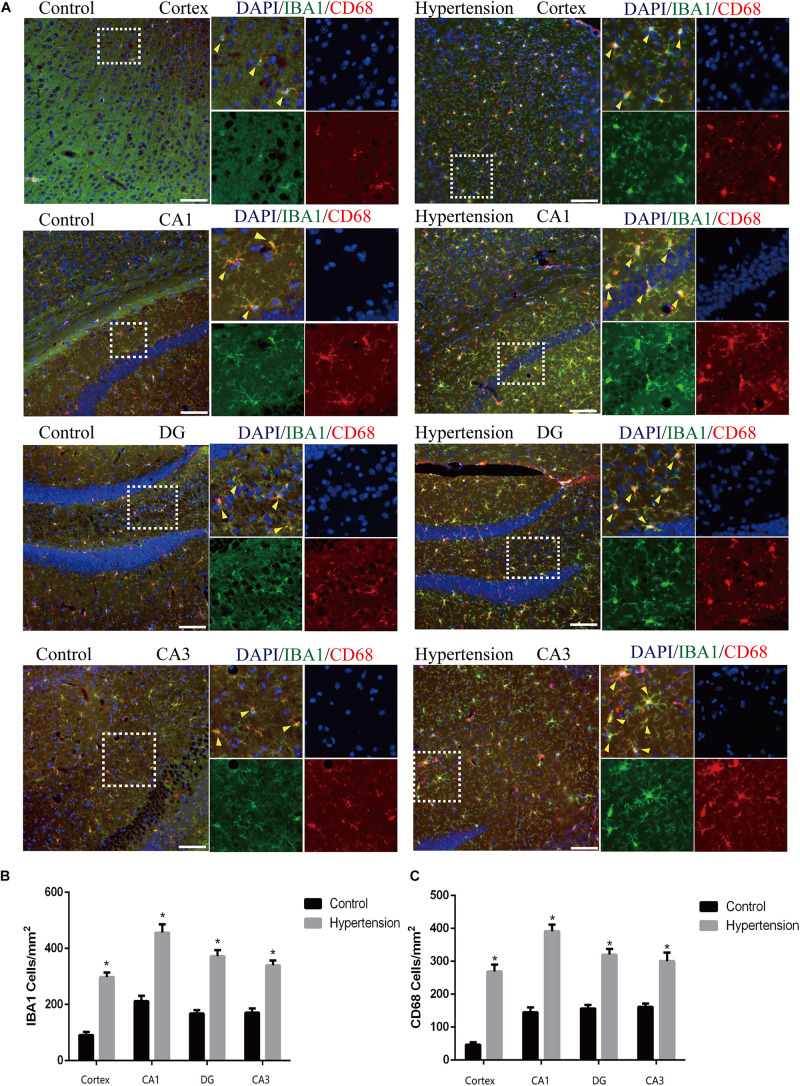
Angiotensin II-induced hypertension upregulates the expression of microglial marker and promotes the activation of M1 microglial transformation in middle-aged mice. **(A)** Representative images of brain slices stained with the anti-IBA1 (green) and anti-CD68 antibodies (red) in the cortex and hippocampus (CA1, DG, and CA3) of mice in the control and hypertension groups (immunofluorescence, ×20, scale bar = 50 μm). **(B)** Histograms comparing the number of IBA1-positive cells in the cortex and hippocampus (CA1, DG, and CA3) of mice in the control and hypertension groups. **(C)** Histograms comparing the number of CD68-positive cells in the cortex and hippocampus (CA1, DG, and CA3) of mice in the control and hypertension groups. Data are expressed as the means ± SD. * *p* < 0.05.

In this study, we used Iba1, a cytoplasmic protein marker of resting microglia (Bennett et al., 2016), and CD68, a lysosomal marker of microglial activation (Bennett et al., 2016), to characterize M1 microglia found in the cortex and hippocampus of mice in each group (Figure 4A). The microglia in the middle-aged mice with hypertension exhibited a more robust expression of CD68 (46.85 ± 8.23 vs. 266.28 ± 20.76, *p* < 0.01, cortex; 145.142 ± 15.61 vs. 389.71 ± 21.02, *p* < 0.01, CA1; 157.71 ± 11.51 vs. 324.57 ± 12.52, *p* < 0.01, DG; 160 ± 8.64 vs. 302.85 ± 27, *p* < 0.01, CA3), and Iba-1 (89.71 ± 9.75 vs. 301.71 ± 12.82, *p* < 0.01, cortex; 213.71 ± 19.43 vs. 452 ± 30.02, *p* < 0.01, CA1; 167.42 ± 13.15 vs. 370.28 ± 22.96, *p* < 0.01, DG; 171.42 ± 16.56 vs. 335.42 ± 12.09, *p* < 0.01, CA3) than control mice with vehicle, and the microglia in the mice with hypertension displayed a highly activated ameboid morphology with large bodies and few thick cellular processes, which indicates microglial activation.

#### Neurotoxic A1 Astrocytic Activation

**FIGURE 5:**
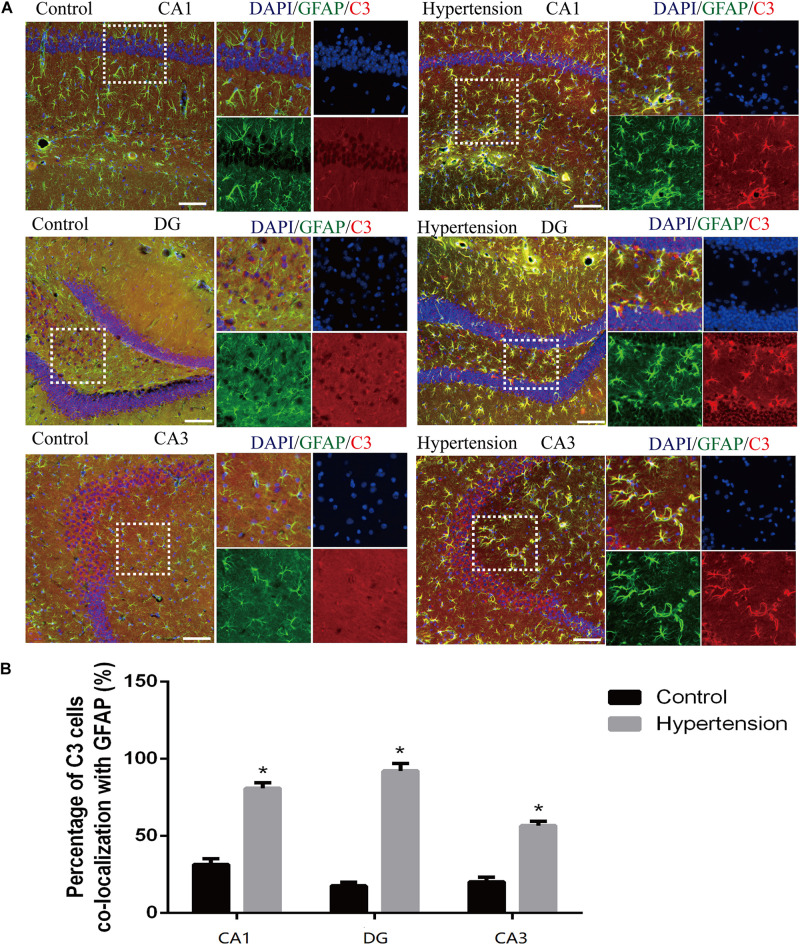
Astrogliosis and notable reactive A1 astrocytes were found in the hippocampus of middle-aged mice after 30 days of angiotensin II treatment. **(A)** Representative images of immunofluorescence double staining with the anti-GFAP (green) and anti-C3 antibodies (red) in the hippocampus (CA1, DG, and CA3) of the mice in the control and hypertension groups (immunofluorescence, ×20, scale bar = 100 μm). **(B)** Histograms comparing C3 levels of astrocytes in the hippocampus (CA1, DG, and CA3) of the mice in the control and hypertension groups. Data are expressed as the means ± SD. * *p* < 0.05.

According to previous research, A1 astrocytes highly upregulate numerous classic complement cascade genes that are destructive to neurons and oligodendrocytes. In particular, complement component 3 (C3) is one of the characteristic genes of A1 astrocytes (Liddelow et al., 2017). To investigate whether A1 astrocytes and C3 participated in neuro-inflammation in the animal models, we measured A1 astrocytes in hippocampal areas (DG, CA1, and CA3) by assessing the anti-GFAP and anti-C3 antibody staining through immunofluorescence. As shown in Figure 5, the reactive astrocytes overexpressing GFAP were found in hippocampal areas (DG, CA1, and CA3) of mice in each group. Furthermore, the A1 astrocytic marker C3 was obviously upregulated in the hippocampal areas of the middle-aged mice with hypertension compared with the very weak expression of C3 in the control group (31.14 ± 4.01% vs. 81.42 ± 3.33%, *p* < 0.01, DG; 17.71 ± 2.42% vs. 92 ± 4.84%, *p* < 0.01, CA1; 21 ± 2.3% vs. 56.71 ± 2.81%, *p* < 0.01, CA3).

#### Spatial Co-localization Between M1 Microglia and A1 Astrocytes

**FIGURE 6:**
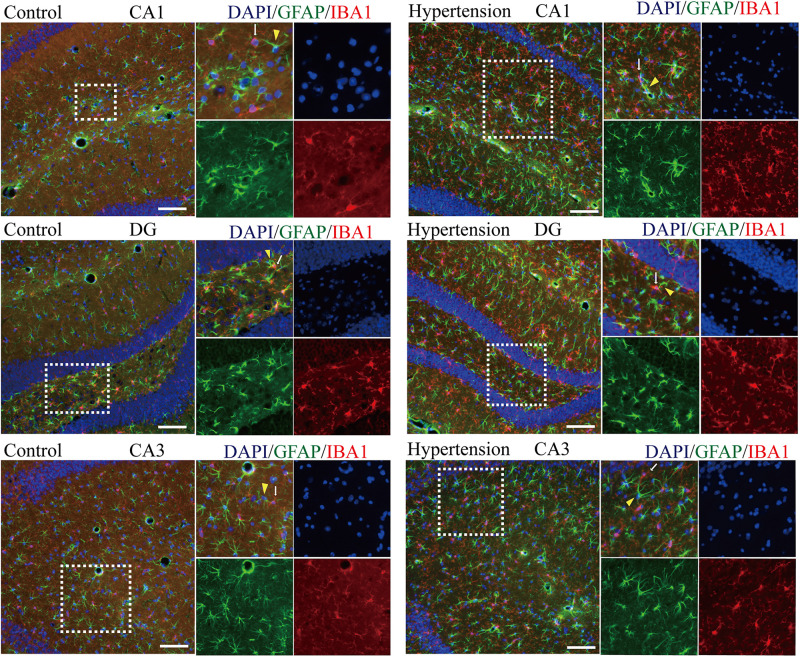
Determination of spatial colocalization of microglia and astrocytes after 30 days of angiotensin II treatment in middle-aged mice. Representative images of immunofluorescence double-staining with anti-GFAP (green) and anti-IBA1 antibodies (red) in the hippocampus (CA1, DG, and CA3) of the mice in the control and hypertension groups (immunofluorescence, ×20, scale bar = 100 μm).

Because M1 microglial activation provokes neurotoxic astrocyte reactivity (Liddelow et al., 2017), we wondered whether there was a specific spatial co-localization promoting a microglia-astroglia interaction. As shown in Figure 6, although there are more activated microglia and reactive astrocytes in the hippocampal areas (DG, CA1, and CA3) of the mice in the hypertension group, their distance and the closeness of the connection between microglial cell branches and astrocytic end feet seem to be similar to those of the control group.

### Microglial TREM2 Upregulation in Middle-Aged Mice With Hypertension

**FIGURE 7:**
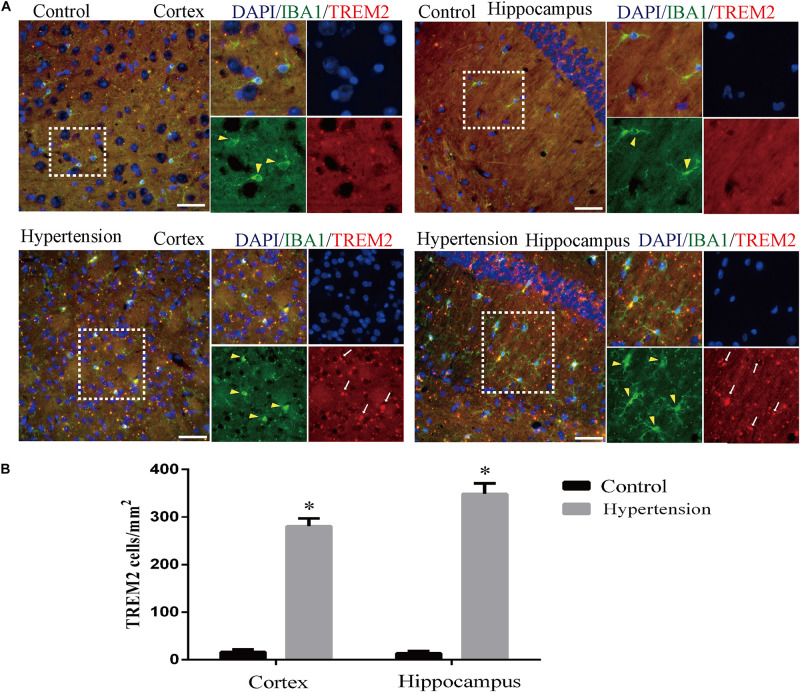
Angiotensin II-induced hypertension increased the expression of triggering receptor expressed on myeloid cells 2 (TREM2) co-localization with microglia marker IBA1 in middle-aged mice. **(A)** Representative images of brain slices stained with the anti-IBA1 (green) and anti-TREM2 antibodies (red) in the cortex and hippocampus of the mice in the control and hypertension groups. **(B)** Histograms comparing the number of TREM2-positive cells in the cortex and hippocampus of mice in the control and hypertension groups (immunofluorescence, ×40, scale bar = 50 μm). Data are expressed as the means ± SD. *p < 0.05.

Furthermore, as revealed in Figure 7, the expression of TREM2 in microglia in the group of mice with hypertension is significantly higher compared with the control group (16 ± 5.65 vs. 280.57 ± 16.56, *p* < 0.01, cortex; 13.42 ± 4.57 vs. 348.57 ± 22.2, *p* < 0.01, hippocampus).

### Microglial TREM2 Overexpression Reversed A1 Astrocytic Activation and Neuronal Toxicity *in vitro*

**FIGURE 8:**
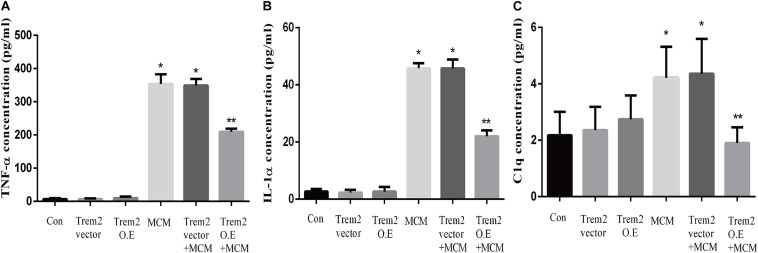
Concentrations of IL-1α, TNF-α, and C1q were tested in MCM, Triggering receptor expressed on myeloid cells 2 (TREM2) (vector or overexpression) transfection-conditioned mediums, and TREM2 (vector or overexpression) + MCM. The concentration levels were measured using ELISA kits. **(A)** Concentration of TNF-α. **(B)** Concentration of IL-1α. **(C)** Concentration of C1q. Differences between the means were determined by *t* test. *p < 0.05 versus control at the match time point, ** *p* < 0.05 versus LPS (0.1 ug/m) at the match time point. The data are expressed as means ± SEM from three independent experiments. C1q: complement component 1q; IL-1α: interleukin 1α; TNF-α: tumor necrosis factor-α.

**FIGURE 9:**
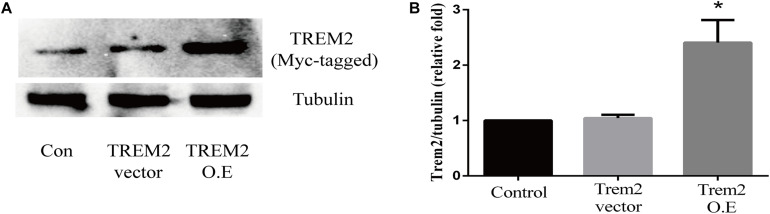
Triggering receptor expressed on myeloid cells 2 (TREM2) protein level was quantitatively measured after TREM2 (vector or overexpression) plasmid transfection in BV2 cells. **(A)** Western blot analysis of TREM2 (Myc-tagged) protein expression. **(B)** Quantitative analysis of TREM2 (Myc-tagged) protein expression. *p < 0.05 vs. control of each protein. The data are expressed as means ± standard deviation (SD) from three independent experiments.

Based on the above histopathological findings, we proposed that the overexpression of microglial triggering receptor expressed on myeloid cells 2 (TREM2) might reverse M1 microglial-induced A1 astrocytic activation and neuronal toxicity. We first incubated the BV2 cells, a microglial cell line, in a serum-free medium containing LPS (0.1 ug/ml) for 24 h to imitate a neuroinflammation *in vitro* environment (Catorce and Gevorkian, 2016; Lopes, 2016). Because the LPS-activated M1 microglia conditioned medium can induce A1 astrocytic activation *via* the secretion of TNF-α, IL-1α, and C1Q *in vitro* (Liddelow et al., 2017), we then performed ELISA to detect the concentrations of TNF-α, IL-1α, and C1Q in the MCM. As shown in Figures 8A–C, the concentrations of TNF-α (6.701 ± 1.323 vs. 353.6 ± 11.84, *p* < 0.05), IL-1α (2.747 ± 0.3313 vs. 45.84 ± 0.7023, *p* < 0.05), and C1Q (2.175 ± 0.3398 vs. 4.206 ± 0.4517, *p* < 0.05) were significantly elevated in the MCM than the concentrations of above cytokines in the normal cell culture media controls. Further, we found that LPS inhibited the TREM2 expression of the BV2 cells in a time-dependent manner (Supplementary Figure 1). To test the hypothesis, we overexpressed TREM2 in the BV2 cells (Figure 9, *F* = 11.32, *p* = 0.0092), which were used for subsequent combination treatments. The results showed that the increase in the above cytokines are alleviated by the overexpression of TREM2 in the BV2 cells [Figures 8A–C (*F* = 673.7, *p* < 0.001), TNF-α; (*F* = 812.8, *p* < 0.001), IL-1α; (*F* = 7.818, *p* = 0.0002), C1Q], but there are no effects on TREM2 in the vector control group. These findings confirmed that TREM2-overexpressing microglia following LPS stimulation did decrease the key chemokines for A1 astrocytic activation.

**FIGURE 10:**
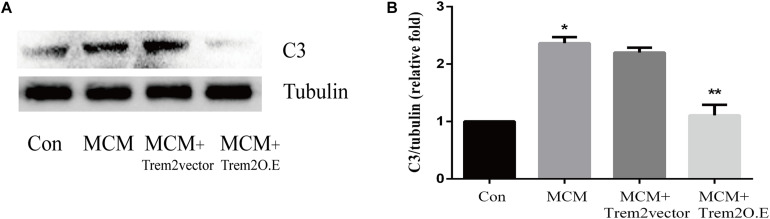
A1 astrocytic marker C3 protein expressions were tested in MCM or triggering receptor expressed on myeloid cells 2 (TREM2) (vector or overexpression) + MCM after incubation for 24 h. **(A)** Western blot of C3 protein; tubulin was used as the internal loading control. **(B)** Quantitative analysis of the C3 protein expression. The data are expressed as means ± standard deviation (SD) from three independent experiments. *p < 0.05 versus control, ** *p* < 0.05 versus MCM. The data are expressed as means ± SD from three independent experiments.

**FIGURE 11:**
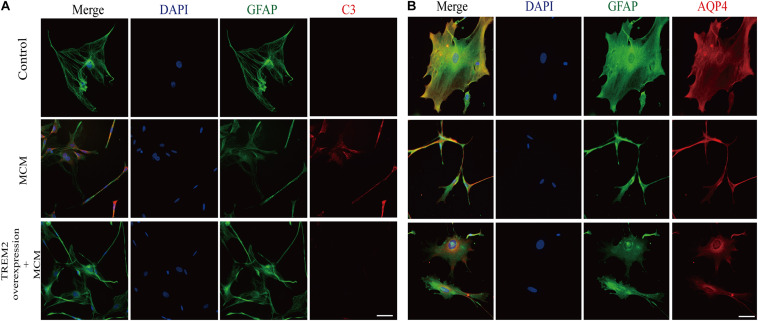
After incubation with MCM or TREM2 overexpression + MCM for 24 h, the astrocytes were subjected to immunofluorescent staining with anti-GFAP (green), A1 anti-C3 (red; **A**), anti-AQP4 (red; **B**), and DAPI (blue) (immunofluorescence, ×40, scale bar = 10 μm).

As indicated in Figure 10, we confirmed that the A1 astrocytic marker C3 expression was significantly increased in MCM than that in control (1 vs. 2.363 ± 0.1056, *p* < 0.05). Afterward, a combination treatment with BV2 microglial TREM2 overexpression and MCM significantly reduced the A1 astrocytic marker C3 expression (2.363 ± 0.1056 vs. 1.107 ± 0.1854, *p* < 0.05), but no significant change in the C3 expression was noted after the TREM2 vector was combined with MCM treatment (Figure 10). Meanwhile, there was no significant change in C3 expression in astrocytes after treatment of LPS or TREM2 (vector or overexpression) transfection conditioned medium, which was served as the negative control (Supplementary Figure 2). Furthermore, immunofluorescence was used to further confirm the results of this study. As shown in Figure 11A, MCM significantly increased the A1 marker C3 expression, which was reversed by BV2 microglial TREM2 overexpression treated with MCM. Meanwhile, we also introduced water channel aquaporin 4 (AQP4), the most prevalent aquaporin channel specifically located at the astrocyte foot processes in the brain parenchyma (Saadoun and Papadopoulos, 2010), to investigate the A1 astrocytes. Figure 11B shows that the foot processes and AQP4 expression were reduced in the A1 astrocytes but rescued by the overexpression of BV2 microglial TREM2 treated with MCM.

**FIGURE 12:**
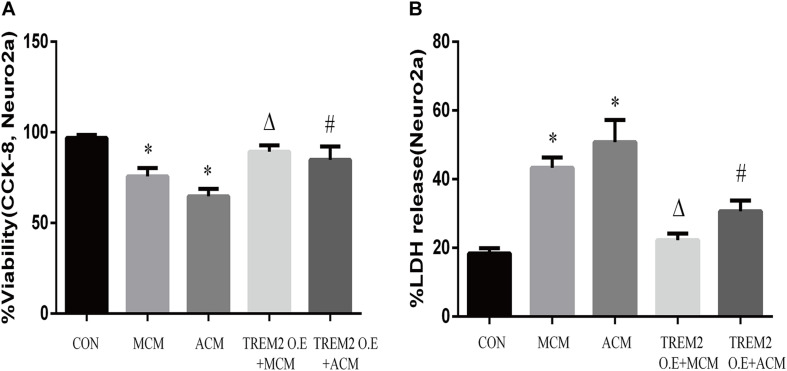
Neuro2A cells were treated with MCM, ACM, triggering receptor expressed on myeloid cells 2 (TREM2) overexpression + MCM, or TREM2 overexpression + ACM, and then CCK-8 and lactate dehydrogenase (LDH) release assays were performed after 24 h. **(A)** CCK-8 assay indicated Neuro2A cells viability changes. **(B)** LDH release assay indicated Neuro2A cells toxicity changes. The data are expressed as means ± SEM from three independent experiments. *p < 0.05 versus control, ^Δ^*p* < 0.05 versus MCM. **^#^***p* < 0.05 versus ACM.

Furthermore, we measured the viability of Neuro2A cells to confirm the findings. As shown in Figures 12A,B, the overexpression of TREM2 in the BV2 cells significantly protect the Neuro2A cells from the toxic effects of MCM and ACM, which are shown as increased cell viability (Figure 12A, *F* = 65.14, *p* < 0.001) and reduced LDH release (Figure 12B, *F* = 129.2, *p* < 0.001).

## Discussion

Alzheimer’s disease consists of two categories, early-onset AD (EOAD, age < 65) and late-onset AD (LOAD, age ≥ 65). EOAD only comprises approximately 5% of AD cases (Mendez, 2017), shows a stronger tendency toward familial inheritance with more aggressive clinical course, and fewer overall comorbidities than LOAD (Smits et al., 2015; Gerritsen et al., 2016). In contrast, LOAD comprises approximately 95% of AD cases with not only genetic risk factors but also medical comorbidities, lifestyle, and other environmental factors contribute to its occurrence (Baumgart et al., 2015). Among the comorbidities, hypertension, a critical vascular risk factor, has been confirmed to cause age-related cognitive impairment and accelerate the development of LOAD by inducing cerebrovascular dysfunction in the early stage (Zlokovic, 2011; Baumgart et al., 2015; Csiszar et al., 2017). A variety of studies elucidated that hypertension causes cerebral microcirculation disorder (capillary rarefaction, blood-brain barrier disruption), neurovascular uncoupling (Csiszar et al., 2017; Hammer et al., 2017), cognitive impairment, Aβ deposition, and CAA (Gentile et al., 2009; Carnevale et al., 2012; Wenzel and Munzel, 2015; Cifuentes et al., 2017). However, the mechanisms of hypertension that cause LOAD are still not fully understood.

In this study, chronic infusion with angiotensin II *via* an osmotic mini pump was able to induce hypertension in middle-aged mice with the mechanism by which the renin-angiotensin system (RAS) regulates blood pressure in the human body (Yang and Xu, 2017), and the results were consistent with those of previous publications (Gentile et al., 2009; Chan et al., 2012; Lu et al., 2015). Hypertension significantly increased amyloid deposition and neuronal apoptosis in middle-aged mice compare with the control middle-aged mice. In addition, we observed that most Aβ40 was deposited in the brain parenchyma (Figure 2A), which is different from previous reports on CAA indicating that the location of Aβ40 deposition is outward of blood vessels (Watts et al., 2014). The difference in the location of Aβ40 deposition between CAA and hypertension may be due to different pathological changes. Although there are damages in vasculature in both cases, hypertension may cause more para-vascular damages and subsequently leads to more Aβ40 depositions in para-vascular tissue. Nevertheless, the accumulation of Aβ40 in the brain can lead to dementia at the end in both cases (Qiang et al., 2017).

In addition to Aβ deposition, we observed that hypertension also induces neuronal death by neuro-inflammation, which is in line with previous evidence that proved ANG II-dependent microglial activation induces neuroinflammation acts *via* cross-talk between central renin-angiotensin system type 1 receptors (AT1R) and toll-like receptor 4 (TLR4) (Biancardi et al., 2016; Liu et al., 2016). We further investigated the roles of neuroinflammation in the mouse models of this study. Consistent with previous studies (Heneka et al., 2010; Carnevale et al., 2012; Cai et al., 2014), activated M1 microglia were markedly upregulated in the cortex and hippocampus of the mice in the hypertension group (Figure 4). Importantly, in addition to more neuronal deaths, we found that the reactive astrocytic phenotype A1 astrocytes significantly increased in the hypertension group (Figure 5). Based on the above observation, we hypothesized that M1 microglia and A1 astrocytes may work collaboratively to induce neuronal death. However, further immunofluorescence did not demonstrate that the spatial associations and connections between M1 microglia and A1 astrocytes were closer or that their end feet engaged in more interactions. Then, we proposed that their communication may be through cytokine secretion.

In this study, we confirmed for the first time that microglial TREM2 was upregulated in the hypertension group. A similar regulatory pattern has been reported in an mouse model with AD, which is deemed to be an anti-neuroinflammatory response to M1 microglia (Wang et al., 2016; Yuan et al., 2016; Yeh et al., 2017). We then hypothesized that the overexpression of microglial TREM2 could not only mitigate microglial inflammatory response, but also had salutary effects on reverse A1 astrocytic activation and neuronal toxicity. To test the hypothesis, we incubated an immortalized mouse microglia-like BV2 cell line in an LPS-containing cell culture medium to mimic a neuroinflammatory environment (Lund et al., 2006). We found that the BV2 microglial cells mimicked primary microglia responses with high fidelity (Elkahloun et al., 2019) and were more tolerable for manipulating TREM2 overexpression. Because angiotensin II induces microglial activation through AT1R interacts with TLR4, and BV2 cells are devoid of AT1 receptor gene expression (Xu et al., 2015), BV2 cells are especially suitable for the present study to elucidate the mechanism focusing on TREM2-mediated microglial anti-inflammatory responses via LPS-ignited TLR4 pathway (Srinivasan et al., 2016; Zheng et al., 2016). In this study, we successfully induced A1 astrocytic activation by LPS-induced M1 microglia (Figure 10), which was consistent with a previous study (Liddelow et al., 2017). Subsequently, the activation of both M1 microglia and A1 astrocytes was obviously alleviated by the overexpression of microglial TREM2 (Figures 9, 10). Normally, astrocytes show a typical “star” cell shape and play an essential role in trophic support for neurons, metabolic regulation, and synaptic transmission (Liddelow and Barres, 2017; Morita et al., 2019). In this study, the A1 astrocytic marker C3 was highly expressed, and the “star” cell shape sharply diminished in A1 astrocytes (Figure 11A). In addition, AQP4, which is expressed in the end-feet of the astrocytes, plays a crucial in maintenance of the blood brain barrier (BBB) (Garwood et al., 2017), and the results show that the foot processes and AQP4 expression were reduced in A1 astrocytes (Figure 11B). However, these phenomena of the AQP4 expression were rescued by the overexpression of microglial TREM2 treated with MCM (Figure 11B). To finally verify the findings, Neuro2A cell viability assays confirmed that the overexpression of TREM2 in BV2 cells could protect Neuro2A cells from the toxic effects of MCM and ACM (Figures 12A,B).

## Conclusion

This present study showed that angiotensin II-induced hypertension significantly exacerbated M1 microglial activation, amyloid deposition, and neuronal apoptosis in middle-aged mice. Compared with previous studies that introduced hypertension in 4- to 12-week-old mice, which is considered young age in mouse, we believed that this middle-age hypertension mouse model shared the same principle of midlife hypertension for late life dementia in humans and is more suitable for investigating the relevant mechanisms in the progression of LOAD. Moreover, previous studies generally only have focused on activated microglial neuroinflammation-induced neuronal lesions, but new evidence has confirmed that activated microglia alone were insufficient to cause neuron death, and a more complicated network, such as A1 astrocytes, through complement components was involved in neuronal lesions and contributed to cognitive decline (Liddelow et al., 2017). Most intriguingly, we confirmed that neurotoxic A1 astrocytes were significantly upregulated in the middle-aged mice with hypertension. The findings propose the concept that just as middle age creates “infertile soil, namely, a vulnerable brain microenvironment, hypertension acts as a “bad seed,” a catalyst that quickly promotes amyloid deposition, neuroinflammation, and microcirculation disorder in the brain. Moreover, Aβ, the core pathogen of AD, acts on and stimulates M1 microglial activation, inducing a more serious neuroinflammation (Bagyinszky et al., 2017; VanItallie, 2017). This feedback creates a “vicious circle” between neuroinflammation and neuronal apoptosis, which eventually leads to late-life dementia. More importantly, microglial TREM2 upregulation was identified in middle-aged mice with hypertension. Contrastingly, it is worthy to point out that LPS inhibited microglial TREM2 expression in a time-dependent manner *in vitro* (Supplementary Figure 1), which was also consistent with previous studies (Gawish et al., 2015; Srinivasan et al., 2016). Then, we further overexpressed microglial TREM2 *in vitro* to confirm the hypothesis, and expanded the dimensions of evidence that TREM2 restrained the neuroinflammatory network *via* “cross-talk” between M1 microglia and A1 astrocytes to protect neurons. The above-mentioned findings implied that microglial TREM2 upregulation might be an anti-neuroinflammation response in middle-aged mice with hypertension. With progress in the “vicious circle,” the anti-neuroinflammatory role of TREM2 will fade away in the late-life phase. Further study will be needed to validate the findings in more details of the current mouse model and address the following questions: what is the relationship between TREM2 and angiotensin II? Does TREM2 participate in the regulation of hypertension-induced cerebral microcirculation dysfunctions, or blood pressure? Is TREM2 an approach to regulate a neurovascular unit or a BBB function through restraining the neuroinflammatory network *via* “cross-talk” between M1 microglia and A1 astrocytes?

## Data Availability Statement

The raw data supporting the conclusions of this article will be made available by the authors, without undue reservation.

## Ethics Statement

This study was approved by the Animal Research Committee of Sun Yat-sen University (Guangzhou, China; Committee Reference Number: SYSU-IACUC-2018-000093).

## Author Contributions

XX performed the experiments. All authors contributed to writing the manuscript and have approved the final version of the manuscript.

## Conflict of Interest

The authors declare that the research was conducted in the absence of any commercial or financial relationships that could be construed as a potential conflict of interest.

## Publisher’s Note

All claims expressed in this article are solely those of the authors and do not necessarily represent those of their affiliated organizations, or those of the publisher, the editors and the reviewers. Any product that may be evaluated in this article, or claim that may be made by its manufacturer, is not guaranteed or endorsed by the publisher.
