# Supplementary material for: Microglial TREM2 Mitigates Inflammatory Responses and Neuronal Apoptosis in Angiotensin II-Induced Hypertension in Middle-Aged Mice
Source: Front Aging Neurosci. 2021 Aug 20;13:716917. doi: 10.3389/fnagi.2021.716917 (PMC8417947; doi:10.3389/fnagi.2021.716917)
Supplement: Supplementary file 1 [file Data_Sheet_1.PDF]

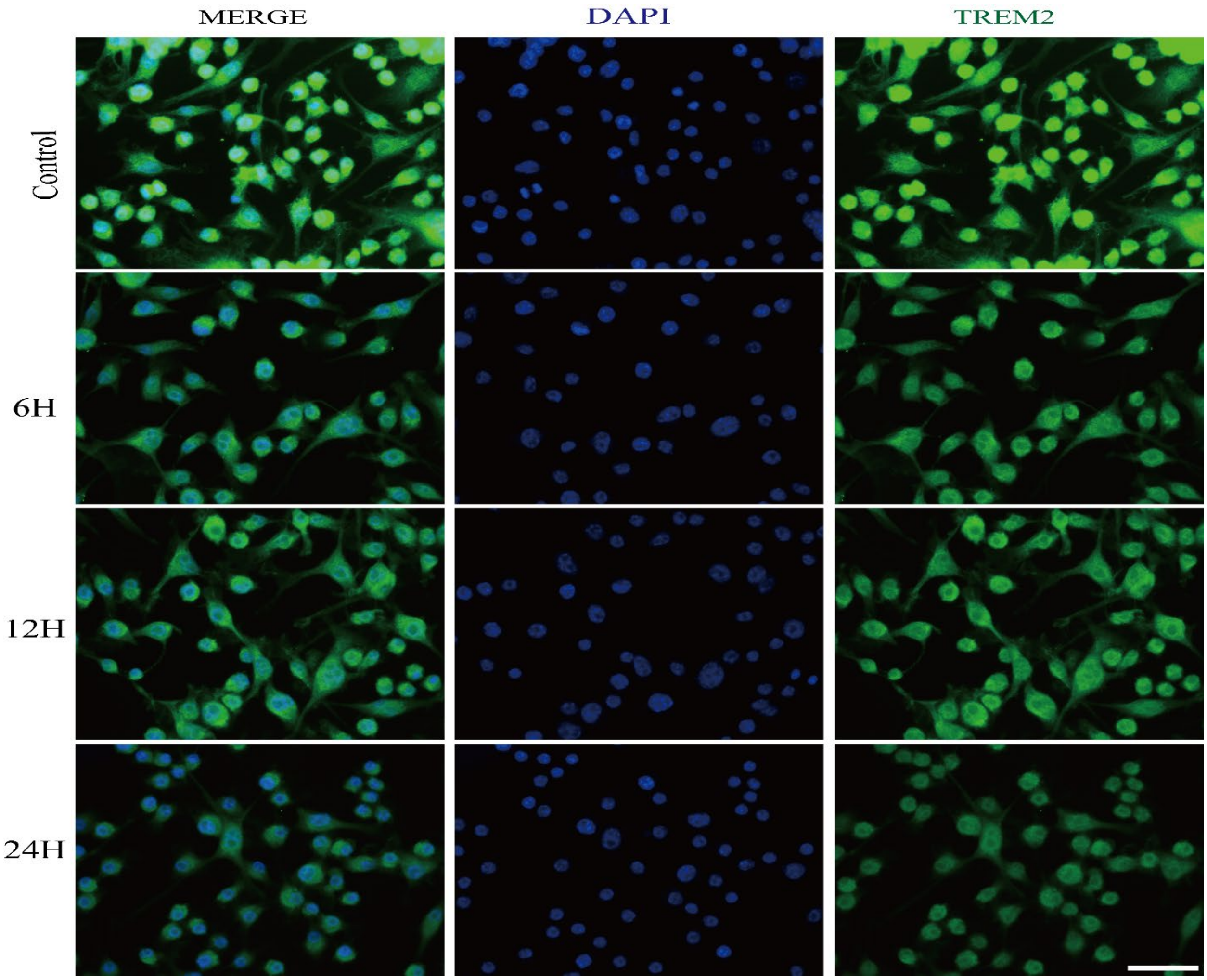

Supplementary figure 1 BV2 microglia cells were incubated with LPS (0.1ug/ml) for the indicated time (6, 12, and 24 hours) to examine the expression of TREM2. BV2 cells were subjected to immunofluorescent staining by anti-TREM2 (green) and DAPI (blue) (immunofluorescence, ×40, scale bar = 50 μm).

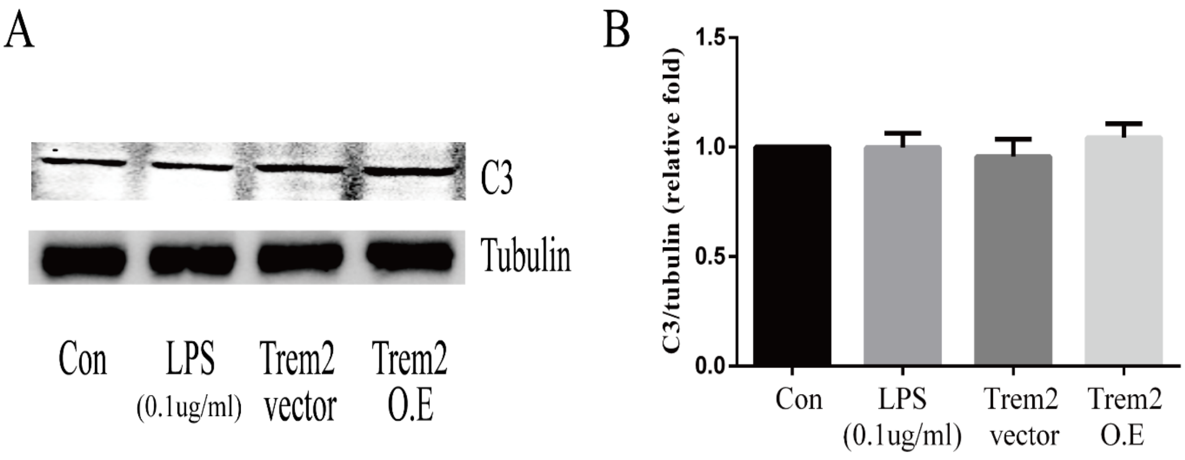

Supplementary Figure 2 Astrocytes were incubated with LPS or BV2 microglial TREM2(vector or overexpression) transfection conditioned medium for 24 hours to examine A1 astrocytic marker C3 protein level. (A) Western blot of C3 protein; Tubulin was used as the internal loading control. (B) Quantitative analysis of C3 protein expression. The data are expressed as means ± SEM from three independent experiments. (F=2.964, P=0.0899)

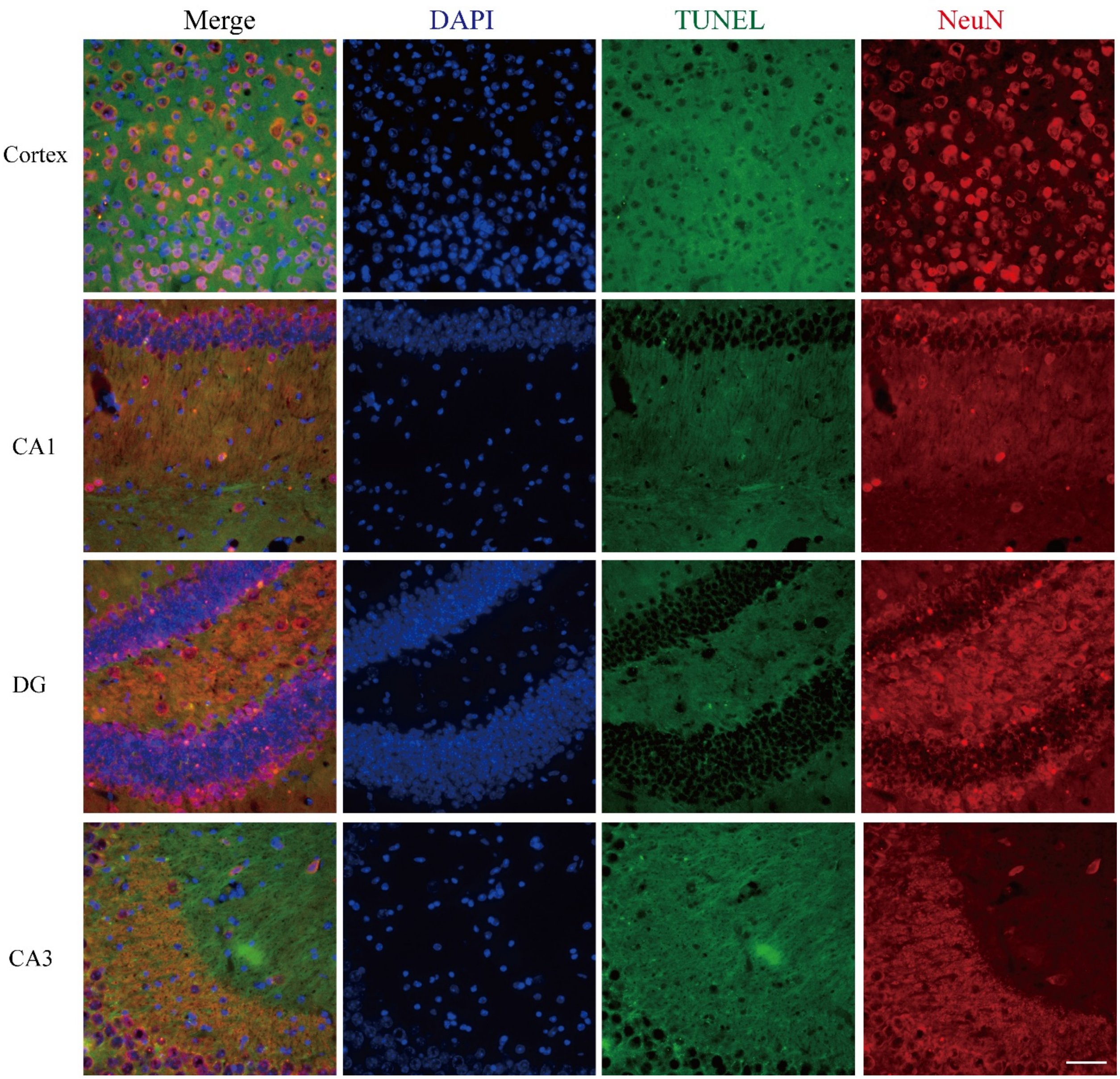

Supplementary Figure 3 Representative negative control images of TUNEL staining assay, which incubate fixed and permeabilized brain sections in Label Solution (without terminal transferase) instead of TUNEL reaction mixture, co-stained with the anti-NeuN antibody (red) in the cortex and hippocampus (CA1, DG, and CA3) of mice (immunofluorescence, ×20, scale bar = 50 μm).

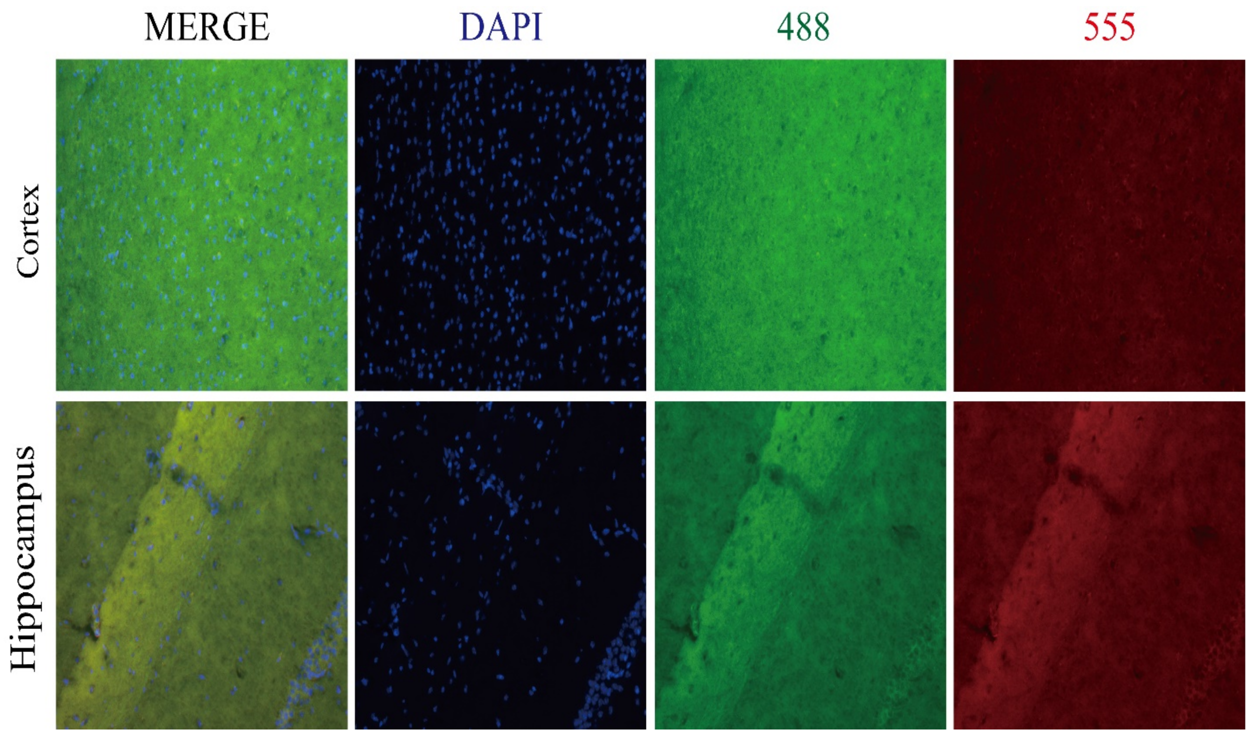

Supplementary Figure 4 Representative negative control images of immunofluorescence double-staining with anti-488 (green) and anti-555 antibodies (red) in the cortex and hippocampus of mice (immunofluorescence, ×20, scale bar = 50 μm).
